# Variable Trust Control Setting for Autonomous Vehicle Highway Navigation and Improved User Experience

**DOI:** 10.1007/s42979-025-03714-x

**Published:** 2025-03-13

**Authors:** James E. Pickering, Jisun Kim, Joshua D’Souza, Keith J. Burnham

**Affiliations:** Aston University, Birmingham, B4 7ET UK

**Keywords:** Control engineering, Autonomous vehicles (AVs), Model-predictive control (MPC), Navigation algorithms, Variable trust control setting (TCS), Vehicle safety

## Abstract

This paper addresses the development of a model-based design approach to enhance the acceptance of safe manoeuvrability of autonomous vehicles (AVs) on highways. A variable trust control setting (TCS) is introduced that empowers users to 'feel in control' of the AV, potentially increasing confidence in, and acceptance of, the technology. This setting is grounded in deontological ethics and utilises virtual boundaries (VBs) to guide driving decisions, i.e., the distance between two AVs interacting with one another. The approach is simulated using a dynamic bicycle model that represents each AV, controlled through an adaptive model-predictive control (MPC) algorithm. The paper outlines the MPC approach, the dynamic bicycle model, and the associated velocity control algorithm. Metrics are introduced to quantify safety of specific AV manoeuvres during interactions with other AVs, enabling the examination of various scenarios. A novel simulation package has been developed to investigate the impact of the proposed variable TCS, focusing on how VBs and steering limitations influence the safety and comfort of AVs during overtaking manoeuvres. The findings demonstrate the effectiveness of this approach, showing that it could potentially allow users to actively manage the safety and comfort aspects of AV operation.

## Introduction

### Literature Review and Outline of Problem

The UK Government has developed a 25-year strategy, detailed in [1], with the aim to foster a greener and more sustainable nation. In 2022, the Government published a document focusing on responsible innovation in autonomous vehicles (AVs), emphasising ethics, safety, and transparency in the development of navigation algorithms, see [2]. Concurrently, the UK is endeavouring to become a leader in the development and implementation of AVs [3]. Such developments have led to the increasing adoption of cyber-physical systems (CPSs) in both AVs and broader transportation system networks. CPSs are complex, multidimensional systems that integrate sensors and actuators through wireless communication networks and computing, thereby connecting the cybernetic and physical environments [4]. This approach shows great potential for improving the safety, environmental sustainability, and efficiency of transportation systems [5].

The above introduces the possibility for a fleet of AVs within which an individual AV can ‘behave’ differently based on a predefined user setting. If there is low trust in AV technology, then the AV does not enter the proximity of other AVs. It is conjectured that this may increase the adoption of the technology. Due to user defined variability, the on-board navigation algorithms will need to be programmed to consider user preferences and factor-in safety and ethical considerations. This will ensure that AV navigation algorithms are justiciable, reasonable and understandable by the users. Simulation models can be used to explore novel navigation algorithms as they are safer and less expensive, see [6] and [7]. Determining how an AV will perform in simulation is a crucial step as it enables different navigation algorithms to be explored and any potential defects to be highlighted and considered at the design stage.

Trust is an important element for users and occupants of AVs due to the likelihood of entering vulnerable situations whereby the users entrust faith in the system [8]. Trust can have an impact on the decision of users to embrace the deployment of such automation [9]. Trust is defined as the “attitude that an agent will help achieve an individual’s goals in a situation characterised by uncertainty and vulnerability” [10]. It is also described as the “willingness of a party to be vulnerable to the actions of another party based on the expectation that the other will perform a particular action important to the trusting party, irrespective of the ability to monitor and control that other party” [11]. For there to be trust in the automation, the multifaceted construct that embraces performance, process and purpose must be established. Performance is related to consistency, stability, and desirability of automation. Process indicates the operators’ knowledge of the underlying algorithms that govern behaviour of the system. Purpose represents the producers’ intention in creating the system [12]. The introduction of AVs is expected to be accompanied by user uncertainty, resulting in varying levels of trust among users. This variability in trust will raise questions about how AVs behave in specific scenarios, such as, for example, executing a lane change to exit at a junction. Consequently, it is anticipated that during the initial phase of AV deployment, user trust levels could differ significantly. This implies the possibility of mismatch between the capability of AVs and users’ expectations that are shapedbased on their previous driving experiences. The mismatch could lead to reduced task effectiveness and efficiency that can be detrimental in the driving context. This warrants the need to calibrate trust in AVs [13]. Hence, it is proposed that trust should be a variable within the design of AV navigation algorithms. Trust in AVs is a crucial aspect which may limit their adoption and integration into society. Trust has been addressed as a factor that needs calibration, see [14] and [15]. The methods utilised for calibrating trust have been used for designing human-machine-interfaces (HMIs). Also, this is to enhance system transparency and to provide information on system capabilities and limitations, see [14] and [15]. However, these approaches do not consider the behaviour of AVs and their related control. Recent literature has highlighted the multidimensional nature of trust, encompassing factors such as reliability, safety, transparency, and user experience. In [16], the authors emphasise the significance of reliability in shaping trust perceptions, as any errors in performance could lead to critical safety issues, thus negatively affecting users’ initial level of trust. However, reliability in this context is contingent upon robust technical infrastructure and effective maintenance protocols. Safety is another fundamental determinant of trust in AVs. Research in [17] indicates that users prioritise safety features and accident prevention mechanisms when assessing their trustworthiness and also when evaluating the potential AV features that would be most attractive to the public. The perceived level of safety is influenced by the vehicle's decision-making processes, collision avoidance strategies, and response to unforeseen circumstances. Transparency emerges as another key factor in fostering trust between users and AVs. In [18], the authors state that gaining proper trust would require transparent communication of AVs' intentions, capabilities, and limitations. Providing users with insights into the vehicle's algorithms, sensor data interpretation, and decision rationale could enhance their understanding and acceptance of autonomous technologies.

### Scenario Considered in this Research

**Fig. 1:**
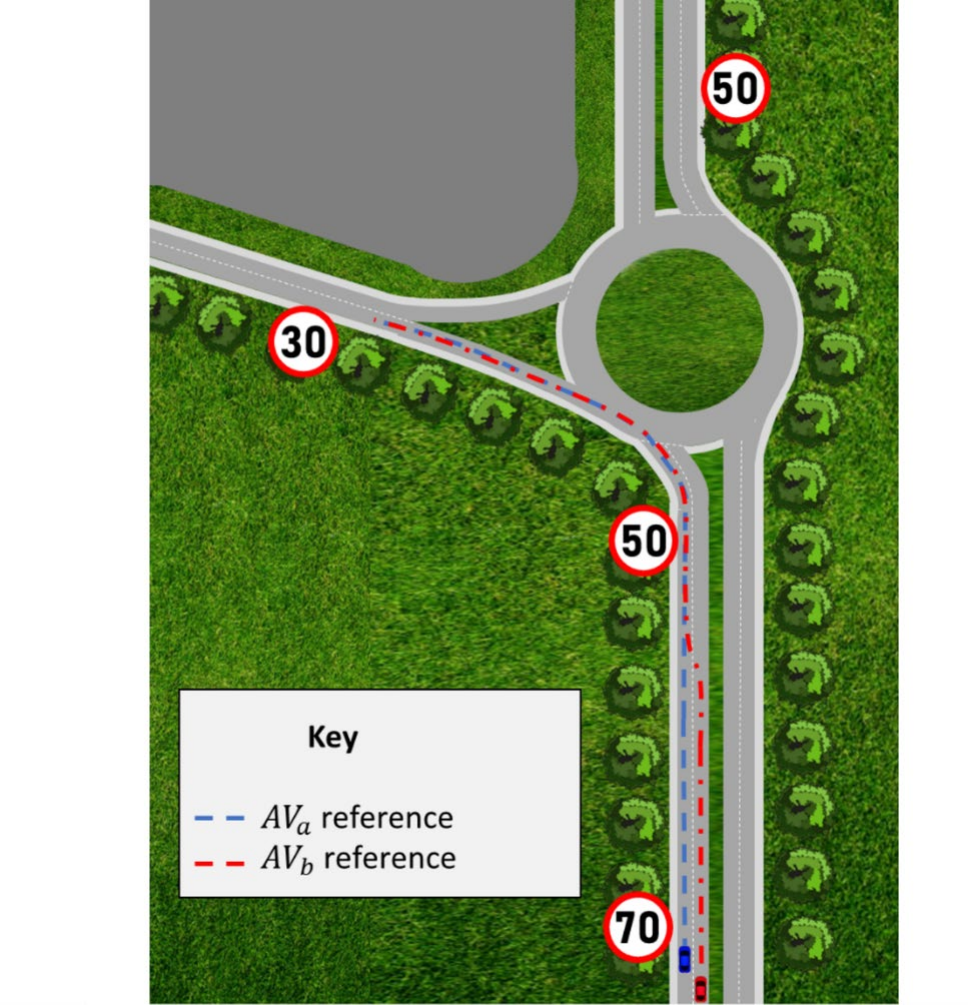
Typical Highway and Roundabout Road Set-Up in the UK

In this research, the scenario involves the use of a two-lane highway, with vehicles on the approach to a roundabout, see Fig. 1. The highway and roundabout considered in this research is representative of a typical configuration in the UK. The highway section model excludes details such as gradient, camber and wheel slip. The highway speed limits shown in Fig. 1 are, 31.29$$m/s$$ (70$$mph$$) on the main stretch of the highway, 22.35$$m/s$$ (50$$mph)$$ as the roundabout is approached and 13.41 $$m/s$$ (30$$mph$$) for the first exit of the roundabout. In Fig. 1, the initial scenario consists of two AVs, denoted $$A{V}_{a}$$ and $$A{V}_{b}$$, for Autonomous Vehicle $$a$$ and Autonomous Vehicle $$b$$, respectively. In this scenario, $$A{V}_{b}$$ is in the right-hand lane and is performing an overtaking manoeuvre on $$A{V}_{a}$$, before both AVs then plan to exit the highway via the first exit of the roundabout. The reference (waypoints) for each of the AVs are given, i.e., blue for $$A{V}_{a}$$ and red for $$A{V}_{b}$$.

To provide a baseline for comparison, as detailed above, the initial problem considers the interaction of the two AVs when performing the overtaking manoeuvre on the approach to a roundabout. The aim is to develop navigation algorithms that consider deontological ethical principles, based on metrics involving safety. This is then used to develop a variable trust control setting (TCS) and to evaluate the effectiveness of the algorithm via a derived safety measure, or metric. Research presented in [19] has involved the development of a simulation package that estimates the collision probability and harm. In this research, a similar approach has been taken in the development of the proposed variable TCS. In [20], a coordinated control approach using model predictive control (MPC) for ethical manoeuvres of AVs is developed – a similar approach is adopted in this research. In addition to this, the effect of passenger comfort will also be investigated, i.e., assessing the effect that an increasing/decreasing passenger comfort has on the variable TCS in an increasing/decreasing manner.

### Hypothesis, Contributions and Outline of Paper

Based on the premise alluded to in Section Literature Review and Outline of Problem, the paper is motivated by the following hypothesis: Incorporation of user defined TCSs into the navigation algorithms of AVs can significantly enhance user trust and adoption rates of the technology, by dynamically adjusting the vehicle's operational behaviour according to individual comfort and safety preferences. These adjustments will lead to measurable improvements in user satisfaction, and overall trust in AV technology. This hypothesis is grounded in the idea that personalising AV behaviour based on user preferences and trust levels, as determined through user settings, will directly impact the perception and effectiveness of AVs in real-world scenarios. By aligning the operational characteristics of AVs with user expectations and ethical considerations, it is anticipated that users will be more inclined to trust and adopt this technology, thus enhancing the overall safety and efficacy of autonomous transportation systems.

The main contributions of this article are as follows:

i. Introduction of a variable TCS that allows users to dynamically control the virtual boundaries (VBs) and maximum steering rate of AVs. This feature uniquely enables users to adjust how the AV operates based on their personal comfort and trust in the technology, offering a customisable user experience.
ii. Empirical investigation into how variations in the trust setting affect risk metrics such as the relative collision metric (RCM) and duration of risk imposed (DRI). This study is among the first to quantitatively link trust variation with measurable safety outcomes in AVs.

The article is organised as follows. The simulation model is formulated in Section Simulation Model, with the simulation results presented and discussed in Section Results and Discussion. Conclusions of the research as well as an outlook are presented in Section Conclusions and Future Outlook

## Simulation Model

**Fig. 2:**
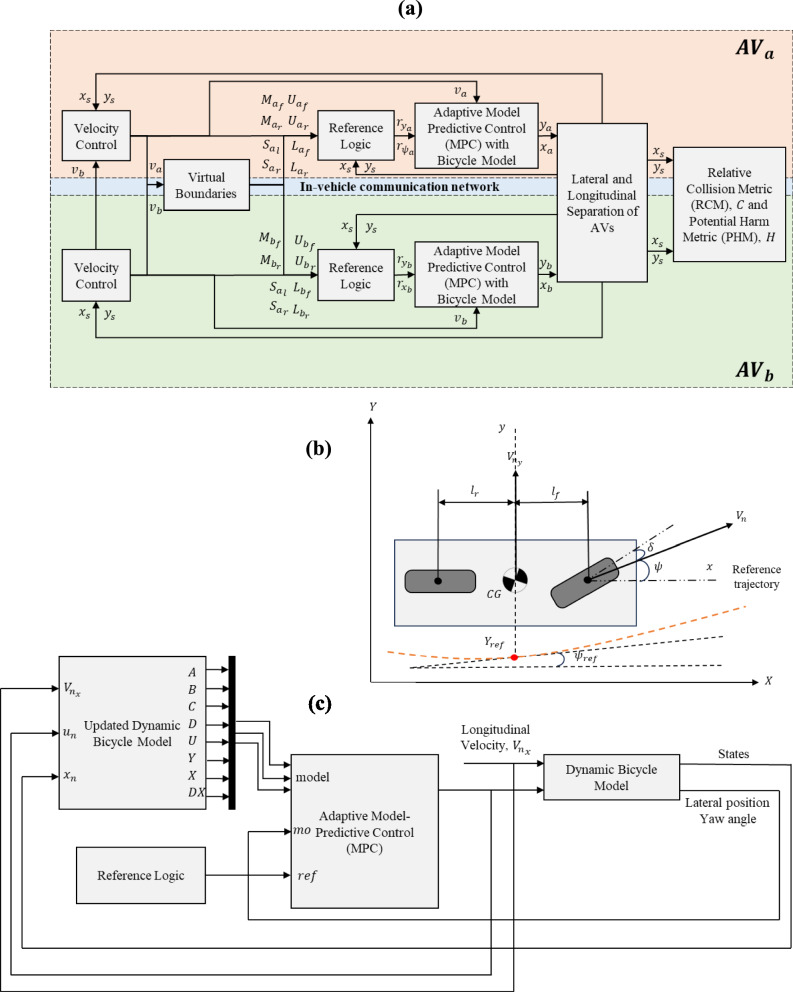
Architecture Framework of the Simulation Model (**a**), Dynamic Bicycle Model (**b**) and Adaptive Model-Predictive Control (MPC) (**c**)

A simulation model considering $A{V}_{a}$ and $A{V}_{b}$ navigation is developed using a CPS approach, see Fig. 2a, with further details given in the following sub-sections.

The simulation model is based on the following initial assumptions:

i. AVs can communicate (e.g., known position and velocity) using vehicle-to-vehicle (V2V) communication.
ii. AVs are equipped with onboard sensors (e.g., cameras) to allow other AVs in each localised vicinity to be identified.
iii. AVs adhere to maximum speed limits of 26.82$m/s$ (60$mph$) in the left-hand lane and 31.29$m/s$ (70$mph$) in the right-hand (overtaking) lane.

### Rule-Based Trajectory Algorithms and Operation of Virtual Boundaries

This section presents the concept of VBs and outlines their application in the design of AV deontological navigation algorithms with a variable TCS, previously developed in [21]. These algorithms expand upon the framework previously established in [22].

Considering the scenario introduced in Section Scenario Considered in this Research, the navigation algorithms are designed for the following situations:

1. Highway lane behaviour (longitudinal control)
2. Lane changing behaviour (lateral control)
3. Entering a decision point (roundabout junction)

#### Virtual Boundaries

**Fig. 3:**
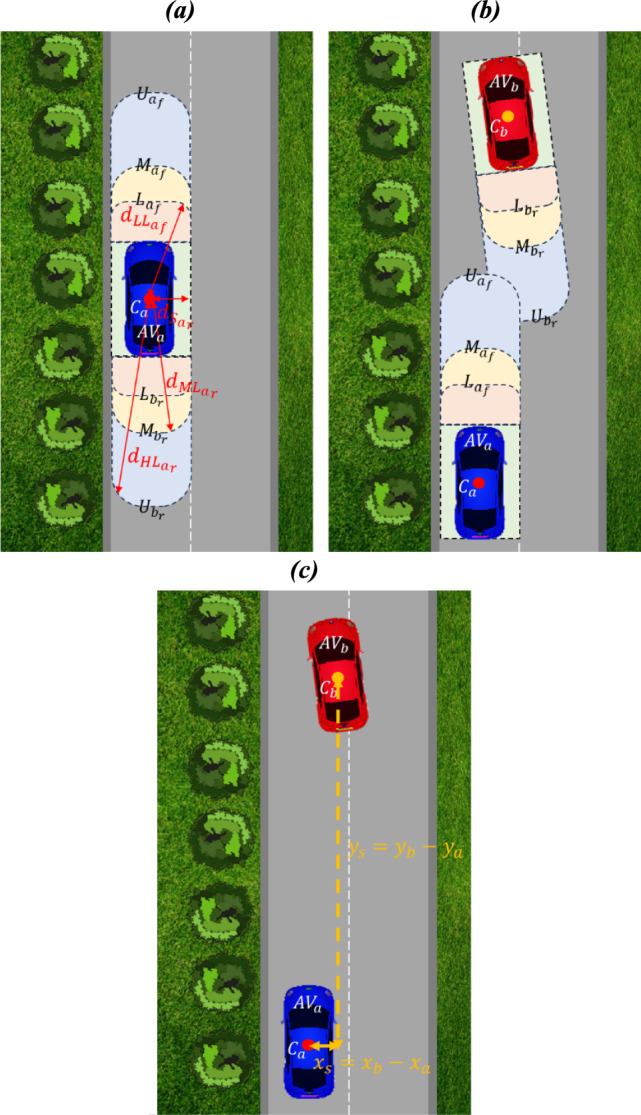
Virtual Boundaries (VBs) for Highway Driving (**a**), VB Overlap for Deontological Algorithm Design when Overtaking (**b**) and Details of how the Separation Distances are Determined (**c**)

The VBs are used to provide a framework for the safe operation of AVs, whilst considering the navigation algorithm design. The VBs represent distances set-up from the centre of each AV, denoted ${C}_{n}$ (where the subscript $n$ denotes the Vehicle ID, i.e., Vehicle $a$ or $b$), to quantify the potential risks, see Fig. 3a.

The VBs to the side of an AV are set-up to ensure that AVs travel in the centre of a given lane, see Fig. 3. The VBs to the side of a vehicle are given by:

- Side denoted ${{S}_{n}}_{l}$ and ${{S}_{n}}_{r}$ for the left and right (where $n$ is the Vehicle ID) of a given vehicle, respectively. Vehicles should avoid entering each other’s ‘Side’ VB. In Fig. 3a, the ${{S}_{a}}_{r}$ distance, denoted $d$, for $A{V}_{a}$ is given, denoted ${d}_{{{S}_{a}}_{r}}$. The value given for both ${{S}_{n}}_{l}$ and ${{S}_{n}}_{r}$ is 2 m, i.e., half the width of the lane.

The VBs to the front and rear of a vehicle, which relate to the speed dependent braking distances, are given by the three-tiered layers:

- Lower Limit (LL) denoted ${{L}_{n}}_{f}$ and ${{L}_{n}}_{r}$ for the front and rear of a given vehicle, respectively. The LL is a VB that must be avoided by any AV – entering this will possibly involve a collision. Whilst unavoidable collisions are not considered here, research in this area may be found in [23] and [24]. In Fig. 3, the ${{L}_{a}}_{f}$ distance, denoted $d$, for $A{V}_{a}$ is denoted ${d}_{{{L}_{a}}_{f}}$.
- Middle Limit (ML) denoted ${M}_{{n}_{f}}$ and ${M}_{{n}_{r}}$ for the front and rear of a given vehicle, respectively. The preferred operation of an AV is to avoid entering this VB. In Fig. 3, the ${M}_{{a}_{r}}$ distance, denoted $d$, for $A{V}_{a}$ is denoted ${d}_{{M}_{{a}_{r}}}$.
- Upper Limit (UL) denoted ${U}_{{n}_{f}}$ and ${U}_{{n}_{r}}$ for the front and rear of a given vehicle, respectively. The UL is a VB that other AVs can manoeuvre into safely. However, the AVs should aim to leave as soon as possible. The UL VB considers a safety margin, i.e., sufficient braking distance that will maintain satisfactory comfort levels to the occupants on-board a given AV, and other AVs involved. In Fig. 3, the ${U}_{{a}_{r}}$ distance, denoted $d$, for $A{V}_{a}$ is denoted ${d}_{{{U}_{a}}_{r}}$.

The initial VBs for this research are given in Table 1. The VBs are based on the stopping distances between two vehicles (these being functions of velocity), recommended by the Department for Transport and the Driver and Vehicle Standards Agency (DVSA) in the UK, see [25].

**Table 1:** Length of Virtual Boundaries (VBs) as Functions of the Autonomous Vehicle (AV) Velocity

Velocity [$mph$]	Virtual Boundaries		
	Lower Limit (LL), ${{L}_{n}}_{f} and$ ${{L}_{n}}_{r}$[$m$]	Medium Limit (ML) ${M}_{{n}_{f}}$ and ${M}_{{n}_{r}}$ [$m$]	Upper Limit (UL) ${U}_{{n}_{f}}$ and ${U}_{{n}_{r}}$ [$m$]
0	$D +$ 0.00	$D+0$.00	$D +$ 0.00
10	$D +$ 1.14	$D +$ 2.29	$D +$ 4.58
30	$D +$ 5.72	$D +$ 11.43	$D +$ 22.86
50	$D +$ 13.34	$D +$ 26.67	$D +$ 53.34
70	$D +$ 24.00	$D +$ 48.01	$D +$ 96.01

As the VB distances are set-up from the centre of each vehicle, the front and rear lengths of the vehicle, denoted $D$, are defined, see Table 1. In this work, $D$ is 1.4 $m$, i.e., so that the overall length of the vehicle is 2.8 $m$. In the simulation model, the UL VB is programmed to emulate the stopping distances, with the ML and LL VBs initially being 50$\%$ and 25$\%$ of the stopping distances, respectively, see Table 1. These VB lengths are further investigated in Section Results and Discussion via an initial sensitivity analysis.

#### Deontological Ethics

In deontological ethics, an action is morally good if it follows a predetermined set of rules or moral values, see [26]. Considering Fig. 1, the AV being overtaken is subject to the actions and/or consequences of the AV performing the overtaking manoeuvre, with both AVs adopting the driving rules (DRs) as follows:

1. Highway lane behaviour: All AVs on the highway should maintain an acceptable distance to the AV ahead, behind and to the sides.
2. Lane changing: Lane changing of a given AV on a highway should be undertaken given there is acceptable space to minimise disruption to other AVs, and to avoid accidents.
3. Entering a decision point: When entering a decision point, an AV should only proceed forward if there are acceptable distances to other AVs ahead or to the sides.

In terms of the VBs, the ‘acceptable distance’ is based on the UL of the two AVs, see Fig. 3b. The DRs have the same maxim applied (a maxim is a rule that connects an action to the reasons for the actions), hence: act in such a way as to minimise impact to other road users (i.e., not effecting another AVs journey time, respecting human lives, and not causing damage to other AVs) and the environment.

#### Variable Trust Control (VTC) Setting

The proposed variable TCS, which is demonstrated in simulation in Section Results and Discussion, is based on the VB lengths given in Table 1, and the maximum steering rate, see Section Sensitivity Analysis involving Maximum Steering Rate.

It is proposed that an operator/user of the AV has an option to select from a range of TCSs. In this paper, five trust settings are considered. These are no trust, little trust, medium trust, medium-to-high-trust and complete trust, see Table 2. In this work it is assumed that the setting can be specified upon entering the AV or adjusted during usage, i.e., the setting can be adjusted if the users trust level of the AV technology changes. A setting of 0% implies no trust in the AV technology and 100% implies complete trust in the AV technology. In this work, the level of trust is associated with a perceived risk, as was considered in [14]. Essentially, varying the level of trust in the AV will result in varying the distances of the VBs or the maximum steering rate.

**Table 2:** Linguistic Terms and how these Correspond to the Variable Trust Control Settings (TCS), $\text{T}$

Trust [%]	Linguistic terms	Variable TCS, *T* [1.20–0.80]
0	No trust	1.20
25	Little trust	1.10
50	Medium trust	1.00
75	Medium to high trust	0.90
100	Complete trust	0.80

The variable TCS values, denoted $T$, for the five settings are given in Table 2. These settings correspond to the user preferences (i.e., no trust, little trust, medium trust, medium-to-high-trust and complete trust). The associated values of variable TCSs, in the range 1.20 to 0.80 are used to adjust the VB lengths, with a medium trust setting of 1.00 corresponding to the nominal VB lengths given in Table 1 implying no adjustment. In the case of complete trust the VB lengths are reduced by a factor of 0.20 and in the case of no trust the VB lengths are increased by a factor of 0.20.

The above initial variable TCSs which affect VB lengths and steering rates are investigated further to assess vehicle comfort and safety via a sensitivity analysis in Section Results and Discussion.

### Control Strategy

This section introduces the dynamic bicycle model for representing AVs within a global coordinate system, focusing on controlling the steering angle to follow a reference trajectory. It also provides an outline of an adaptive model predictive control (MPC) algorithm that optimises real-time trajectories and a velocity control algorithm that regulates vehicle speeds based on interactions, ensuring safe navigation. In addition, the section introduces metrics for assessing collision risk and potential harm.

#### Dynamic Bicycle Model

A dynamic bicycle model is used to represent each of the AVs, within a global position given by $X$ and $Y$, see Fig. 2b. The vectors from the bicycle model, denoted ${v}_{x}$ and ${v}_{y}$ represent the longitudinal and lateral velocity, respectively. The vehicle’s longitudinal acceleration is assumed to be in a quasi-steady state, i.e., gradual longitudinal acceleration changes resulting in the approximation of steady state responses at each instantaneous state [27]. The aim of the dynamic bicycle model is to follow a reference trajectory denoted ${Y}_{ref}$, given by the red circle on the orange line in Fig. 2b. The reference trajectory is obtained by controlling the steering angle, denoted $\delta$. The yaw angle output, denoted $\psi$, and reference yaw angle, denoted ${\psi }_{ref}$, are determined with reference to the horizontal axis. On this basis, the dynamic model is initially adopted from [28] and is given by:

1 $$\frac{d}{dt}\left[\begin{array}{c}\dot{y}\\ \psi \\ \dot{\begin{array}{c}\psi \\ y\end{array}}\end{array}\right]=\left[\begin{array}{cccc}-\frac{2{C}_{f}+2{C}_{r}}{m{V}_{x}}& 0& -{V}_{x}-\frac{2{C}_{f}{l}_{f}-2{C}_{r}{l}_{r}}{m{V}_{x}}& 0\\ 0& 0& 1& 0\\ \frac{2{l}_{f}{C}_{f}-2{l}_{r}{C}_{r}}{{I}_{z}{V}_{x}}& 0& -\frac{2{l}_{f}^{2}{C}_{f}+2{l}_{r}^{2}{C}_{r}}{{I}_{z}{V}_{x}}& 0\\ 1& 0& 0& 0\end{array}\right]\left[\begin{array}{c}\dot{y}\\ \psi \\ \dot{\begin{array}{c}\psi \\ y\end{array}}\end{array}\right]+\left[\begin{array}{c}\frac{2{C}_{f}}{m}\\ 0\\ \frac{2{l}_{f}{C}_{f}}{{I}_{z}}\\ 0\end{array}\right]\delta$$

where ${V}_{x}$ denotes the longitudinal velocity at the vehicle’s centre of gravity (CoG), $m$ denotes the vehicle mass, ${I}_{z}$ denotes the vehicle’s yaw moment of inertia, ${I}_{f}$ denotes the longitudinal distance from the CoG to the front tyres, ${I}_{r}$ denotes the longitudinal distance from the CoG to the rear tyres, ${C}_{f}$ denotes the cornering stiffness of the front tyres, ${C}_{r}$ denotes the cornering stiffness of the rear tyres, $\delta$ denotes the vehicle’s input front steering angle and $y$ denotes the vehicle’s lateral position output. Simulation model parameters are given in Table 3, including maximum acceleration and deceleration values, denoted ${a}_{max}$ and ${a}_{min}$, respectively.

**Table 3:** Simulation Model Parameter Values

Model parameter	Value [units]
${C}_{f}$	19,000 [$N/rad$]
${C}_{r}$	22,000 [$N/rad$]
${m}_{n}$	2400 [$kg$]
${v}_{n}$	0 to 31.29 [$m/s$]
${l}_{f}$	1.2 [$m$]
${l}_{r}$	1.6 [$m$]
${I}_{z}$	1575 [$kg {m}^{2}$]
$r$	140 $[Ns/m]$
${a}_{max}$	$10 [m/{s}^{2}]$
${a}_{min}$	-6 $[m/{s}^{2}]$

Considering Eq. (1), the state space model does not account for the global lateral position as a state variable, denoted $Y$. Therefore, this is determined using the following [29]:

2 $$\dot{Y}=\dot{x}\text{sin}\psi +\dot{y}\text{cos}\psi$$

Using small angle approximations i.e., $\text{sin}\psi \approx \psi$ and $\text{cos}\psi \approx 1$, and considering the notation ${V}_{x}=\dot{x,}$ Eq. (2) can be rewritten as follows:

3 $$\dot{Y}={V}_{x} \psi +\dot{y}$$

The state space model for the 2 degrees of freedom is then given by:

4 $$\frac{d}{dt}\left[\begin{array}{c}\dot{y}\\ \psi \\ \dot{\begin{array}{c}\psi \\ Y\end{array}}\end{array}\right]=\left[\begin{array}{cccc}-\frac{2{C}_{f}+2{C}_{r}}{m{V}_{x}}& 0& -{V}_{x}-\frac{2{C}_{f}{l}_{f}-2{C}_{r}{l}_{r}}{m{V}_{x}}& 0\\ 0& 0& 1& 0\\ \frac{2{l}_{f}{C}_{f}-2{l}_{r}{C}_{r}}{{I}_{z}{V}_{x}}& 0& -\frac{2{l}_{f}^{2}{C}_{f}+2{l}_{r}^{2}{C}_{r}}{{I}_{z}{V}_{x}}& 0\\ 1& {V}_{x}& 0& 0\end{array}\right]\left[\begin{array}{c}\dot{y}\\ \psi \\ \dot{\begin{array}{c}\psi \\ Y\end{array}}\end{array}\right]+\left[\begin{array}{c}\frac{2{C}_{f}}{m}\\ 0\\ \frac{2{l}_{f}{C}_{f}}{{I}_{z}}\\ 0\end{array}\right]\delta$$

Using the dynamic bicycle model, $\dot{X}$ is derived from the following equation of motion, see [29]:

5 $$\dot{X}=\dot{x}\text{cos}\psi -\dot{y}\text{sin}\psi$$

Using small angle approximations, and considering the notation ${V}_{x}=\dot{x}$, Eq. (5) can be rewritten as follows:

6 $$\dot{X}={V}_{x}-\dot{y}\psi$$

#### Model-Predictive Control

An adaptive model-predictive control (MPC) algorithm is developed for each AV, see Fig. 2c. MPC is a predictive control method that utilises a dynamic model of the system to optimise future trajectories, enabling the vehicle to make informed decisions in real-time. MPC is able to take into account constraints on control action and system dynamics, making it particularly well-suited for the inherently dynamic nature of AV navigation, see [30] and [31].

By predicting future system states using a mathematical model and optimising control inputs to minimise deviations from a given desired reference trajectory, while respecting constraints, MPC is able to steer the AV towards the desired trajectory. At each time-step, an optimisation problem is solved to determine the appropriate control action, with the process continuously repeating using updated state information. This continuous prediction, feedback and adaptation ensures that the AV effectively follows the desired path.

The MPC algorithm was tuned manually to produce an acceptable value for the integral of absolute error (IAE) by adjusting the control and prediction horizons to be 3 and 20, respectively. The bicycle model adopted here for each of the vehicles is constructed based on the references (waypoints) given in Fig. 1. The constraints are: maximum steering of 30 degrees and for driving comfort the rate of change of steering angle is limited to 15 degrees/second. Further details of the MPC algorithm used in this research can be found in [32].

#### Velocity Control

**Fig. 4:**
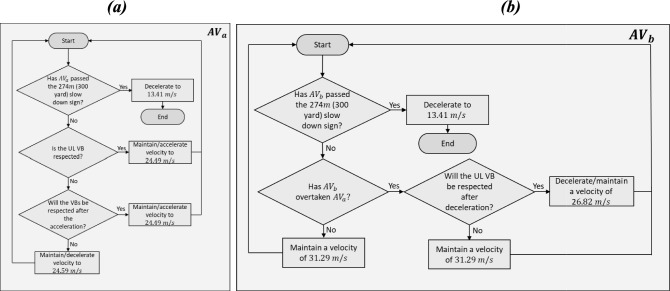
Velocity Control Algorithm for ${\text{AV}}_{\text{a}}$ with a Deontological Navigation Algorithm (**a**), ${\text{AV}}_{\text{b}}$ with a Deontological Navigation Algorithm (**b**)

In a scenario as in Fig. 1, the velocities of $AV_{a}$ and $AV_{b}$ would be influenced by the presence of other vehicles, therefore velocity control is required. For $AV_{a}$*,* the aim of the deontological algorithm (see Section Deontological Ethics) is to maintain a velocity of 26.82 $m/s$(60 $mph$) and to decelerate to 13.41 $m/s$ (30 $mph$) when 274 $m$ away from the roundabout. If at any point in time, the VB UL is not respected (i.e., from another vehicle overtaking), *AV*_*a*_ is programmed to decelerate to a lower velocity of 24.59 $m/s$ (55 $mph$), see Fig. 4a. This velocity is held until the VB UL is again respected by other vehicles. Regarding the velocity control of $AV_{b}$ with the deontological algorithm, an initial velocity of 31.29 $m/s$(70 $mph$) is held while overtaking $AV_{a}$ in Lane 1 with the VB UL being respected, see Fig. 4b. Once this condition is met, $AV_{b}$ will then decelerate to the driving speed of 26.82 $m/s$ (60 $mph$) in Lane 1. In a similar manner to $AV_{a}$*,*$AV_{b}$ will decelerate to 13.41 $m/s$(30 $mph$) as the roundabout is approached whilst constantly respecting the VBs.

#### Reference Logic

**Fig. 5:**
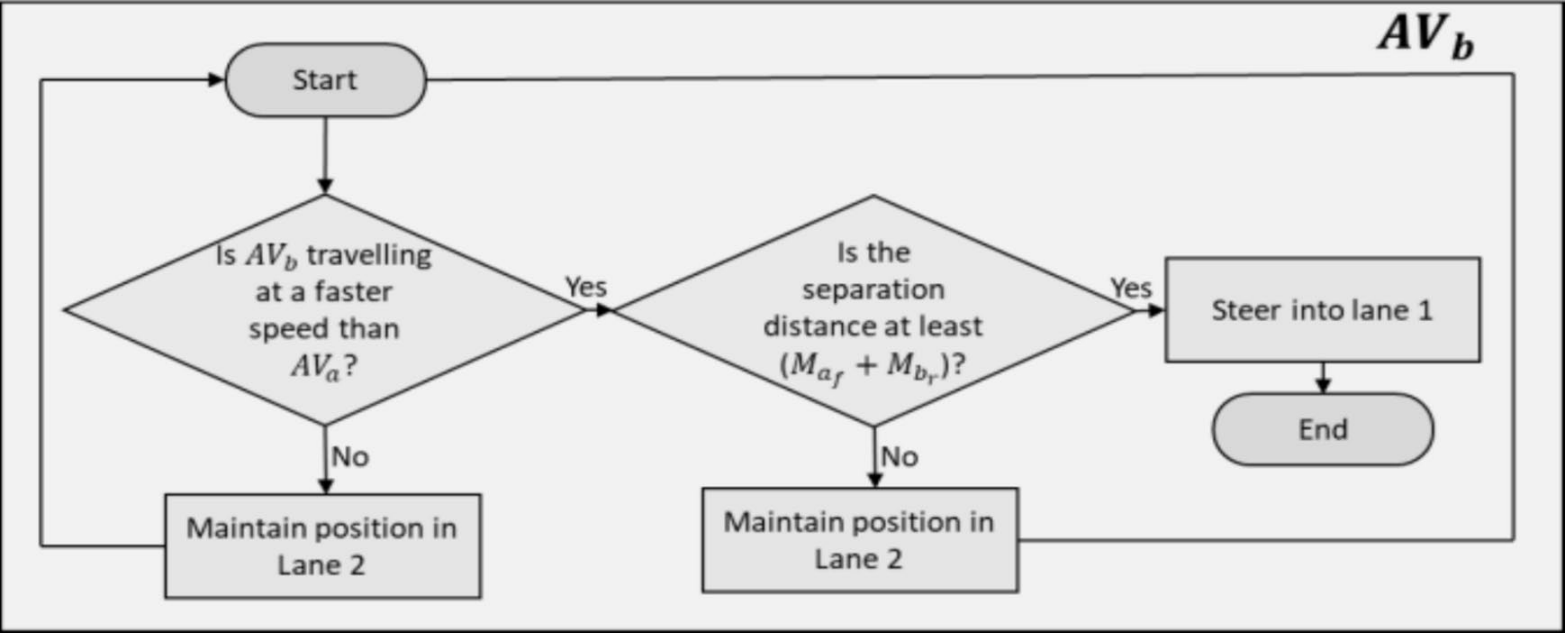
Reference Logic for $A{V}_{b}$

From Fig. 1, $AV_{a}$ must remain in Lane 1 for taking the 1st exit at the roundabout. Similarly, $AV_{b}$ also aims to take the 1st exit. Hence, decision making algorithms are required to account for the lane changing manoeuvre of $AV_{b}$ into Lane 1. Because $AV_{b}$ is programmed to follow the DRs, it is permitted to change lane only if the ML VBs of the two AVs are respected, see Fig. 5.

### Relative Collision and Potential Harm Metrics

The lateral and longitudinal separation distances between two vehicles, as illustrated in Fig. 3c, are given, respectively, by:

7 $${y}_{s}={y}_{b}-{y}_{a}$$

8 $${x}_{s}={x}_{b}-{x}_{a}$$

where ${y}_{s}$ and ${x}_{s}$ denote the lateral and longitudinal separation distances between the vehicles, ${y}_{a}$ and ${x}_{a}$ denote the lateral and longitudinal position of $A{V}_{a}$ at time $t$, and ${y}_{b}$ and ${x}_{b}$ denote the lateral and longitudinal positions of $A{V}_{b}$, respectively.

Equations (7) and (8) are used with the VBs to quantify the risk presented between the two vehicles. The RCM, denoted $C$, provides an approach to quantifying risk and is given by:

9 $$C= \left[1- \frac{{x}_{s}}{{{U}_{n}}_{f}+ {U}_{{n}_{r}}}\right] \left[1- \frac{{y}_{s}}{{S}_{{n}_{l}}+{S}_{{n}_{r}}}\right]$$

Equation (9) outputs values between 0 and 1. A value 0 indicates zero risk, (no overlap of the VBs) hence, no risk of a collision. A value tending towards 1 indicates an increasing risk. If a value of 1 is recorded for the RCM, this is likely to result in a collision.

The potential harm metric (PHM), denoted $H$, provides an approach to quantifying risk and is given by:

10 $$H=\pm \left[\frac{\Delta {v}_{n}}{\Delta {v}_{{max}_{n}}}\right]$$

where $\Delta {v}_{n}$ denotes the difference in the velocities of two vehicles, $\Delta {v}_{{max}_{n}}$ denotes the maximum velocity difference between two vehicles and $\pm$ is used to denoted direction, i.e., positive for vehicles moving towards each other, negative for vehicles moving apart and a value of zero for vehicles travelling at the same velocities (i.e., identical magnitude and direction). The difference in vehicle velocities is commonly used for estimating harm, see [33] and [34]. Equation (10) provides a numerical value to determine risk, i.e., between 0 (no risk) and 1 (high risk).

## Results and Discussion

The results are now detailed for the deontological algorithm in Section Deontological Algorithm (Nominal case with TCS, ), as well as two initial sensitivity study analyses in Section Sensitivity Analysis involving Variable Trust Control Setting (TCS) for a variable TCS, and in Section Sensitivity Analysis involving Maximum Steering Rate for a maximum steering rate.

### Deontological Algorithm (Nominal case with TCS, ${\varvec{T}}=1.00$)

**Fig. 6:**
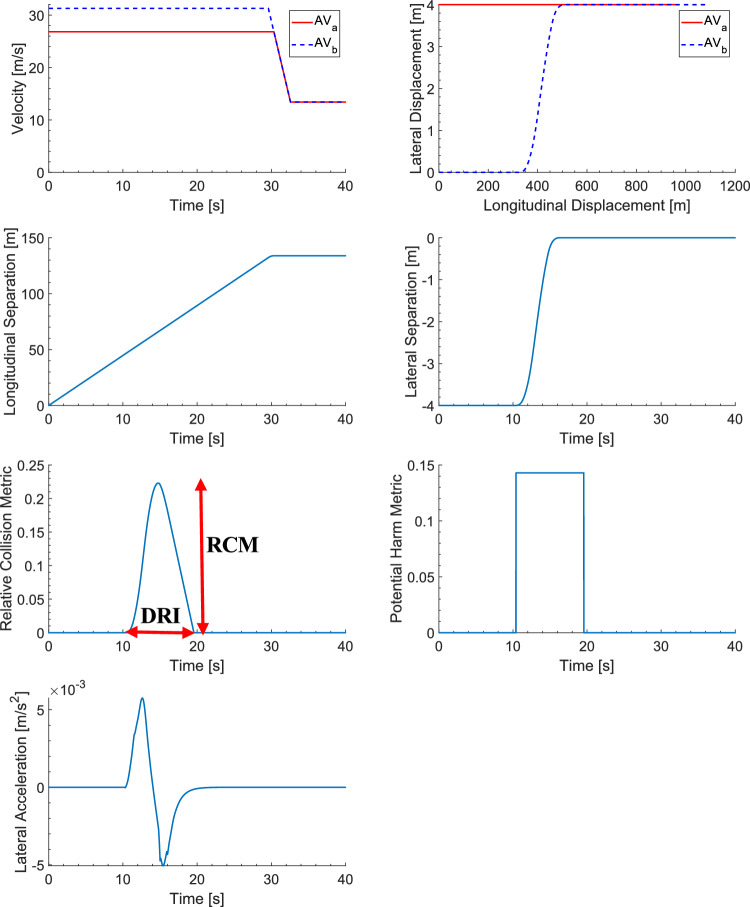
Deontological Algorithm Design Results

Simulation results for the deontological algorithm (as detailed in Section Deontological Ethics) corresponding to the nominal case of medium trust, with a TCS value of unity, are provided in Fig. 6. The graphical output of velocity versus time for the two AVs is given in Fig. 6 (Upper-Left). Recall from Fig. 1, *AV*_*a*_ is travelling in the left-hand-lane, initially at the maximum velocity (i.e., 60*mp*ℎ or 26.82*m*/*s*). *AV*_*b*_ is travelling at the maximum speed in the right-hand-lane, i.e., 31.29*m*/*s* (70*mp*ℎ). At approximately 30s, *AV*_*b*_ performs an overtaking manoeuvre on *AV*_*a*_, with both AVs then travelling at their maximum speeds, i.e., in the left-hand- lane, 26.82* m*/*s* and in the right-hand lane 31.29*m*/*s*. The speed of both AVs (with *AV*_*b*_ now being ahead of *AV*_*a*_) then decreases as the speed limit approaching the roundabout sets-in, i.e., 13.41*m*/*s* (30*mp*ℎ). The lateral displacement versus longitudinal displacement graphical output for *AV*_*a*_ and *AV*_*b*_ is initially given, see Fig. 6 (Upper-Right). Note that the overtaking manoeuvre takes place at around 350*m*. The graphical outputs for the longitudinal and lateral separation of the two AVs are given in Fig. 6 (Upper-Middle-Left and Upper-Middle-Right, respectively). Note that the longitudinal separation distance remains constant once *AV*_*b*_ has completed the overtaking manoeuvre on *AV*_*a*_. The graphical outputs for RCM and PHM for the two AVs are given in Fig. 6 (Lower-Middle-Left and Lower-Middle-Right, respectively). For the RCM, the peak RCM and DRI are labelled, with values of 0.22 and 9.15s being captured, respectively (see Table 4). Both values are relatively low as the rules within the deontological algorithm were respected. The RCM is high for a short duration (approximately 9.15s) due to the velocity control applied to $A{V}_{b}$, resulting in $A{V}_{b}$ travelling at a velocity of 70*mp*ℎ when overtaking *AV*_*a*_, and returning to the desired velocity of 60*mp*ℎ once $A{V}_{a}$ has been overtaken. The PHM during the same time-period is also relatively low, with a positive value of 0.14, see Table 4. Finally, the lateral acceleration of the overtaking vehicle (i.e., $A{V}_{b}$) is given, see Fig. 6 (Lower-Left), with the values for the peak and positive mean acceleration recorded in Table 4. Note that a first order filter was applied to the acceleration versus time data with a filter transfer function of:

**Table 4:** Deontological Algorithm Design Results (Nominal Case, where $T=1.00$*)*

	Maximum Relative Collision Metric (RCM),$C$	Maximum Potential Harm Metric (PHM),$H$	Duration of Risk Imposed (DRI) [Seconds]	Peak Acceleration ${a}_{peak} [m/s]$	Positive Mean Acceleration ${a}_{mea{n}_{p} } [m/s]$
*T* = 1.00 (Medium Trust)	0.22	0.14	9.15	0.0056	0.0037

11 $$G\left(s\right)=\frac{1}{0.1s+1}$$

### Sensitivity Analysis involving Variable Trust Control Setting (TCS)

**Fig. 7:**
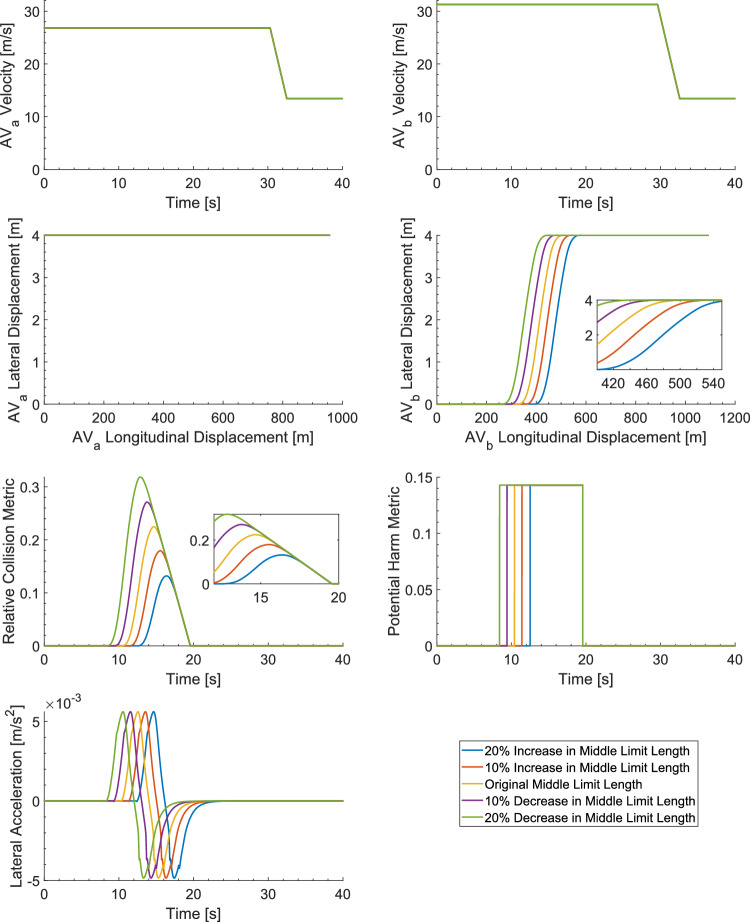
Sensitivity Analysis Results for the Variable Trust Control Setting (TCS)

The results of the sensitivity analysis for the variable TCS, as detailed in Section Variable Trust Control Setting, are presented in Fig. 7. The model has been used to examine the impact of variable TCSs, involving adjustments to the ML length of the deontological AV’s VBs, as shown in Table 2 of Section Variable Trust Control Setting. Figure 7 displays the RCM outcomes across various trust settings, with the PHM and DRI values being given in Table 5. As expected, if the level of trust increases, resulting in lower TCSs, hence smaller ML VBs, then the occupants’ risk increases. Conversely, lower values of trust, leading to higher TCSs, leading to a larger ML VBs, then the occupants risk decreases. Transitioning the trust setting from 'no trust' to 'complete trust' leads to a 19% increase in the RCM and a 22% increase in the DRI. Thus, the 'complete trust' setting exposes occupants to higher levels of risk over a longer duration. Hence, the more confident the user is with the technology, the more risks the user is prepared to take. This will ironically lead to a greater number of risk takers as the technology matures, with this leading to reduced safety. As anticipated, the peak PHM remains constant, due to the AVs' effective velocity control system. This ensures compliance with velocity limits during overtaking and maintaining lane discipline in both the left-hand and right-hand lanes. This result indicates that the peak acceleration values remain constant. Note that Row 3 in Table 5 corresponds to the results presented in Table 4.

**Table 5:** Sensitivity Analysis Results for the Variable Trust Control Setting (TCS)

	Maximum Relative Collision Metric (RCM),$C$	Maximum Potential Harm Metric (PHM),$H$	Duration of Risk Imposed (DRI) [Seconds]	Peak Acceleration ${a}_{peak} [m/s]$	Positive Mean Acceleration ${a}_{mea{n}_{p} } [m/s]$
1.20 (No Trust)	0.13	0.14	7.05	0.0056	0.0037
1.10 (Little Trust)	0.18	0.14	8.15	0.0056	0.0037
1.00 (Medium Trust)	0.22	0.14	9.15	0.0056	0.0037
0.90 (Medium-to- High-Trust)	0.27	0.14	10.15	0.0056	0.0037
0.80 (Complete Trust)	0.32	0.14	11.15	0.0056	0.0037

### Sensitivity Analysis Involving Maximum Steering Rate

**Fig. 8:**
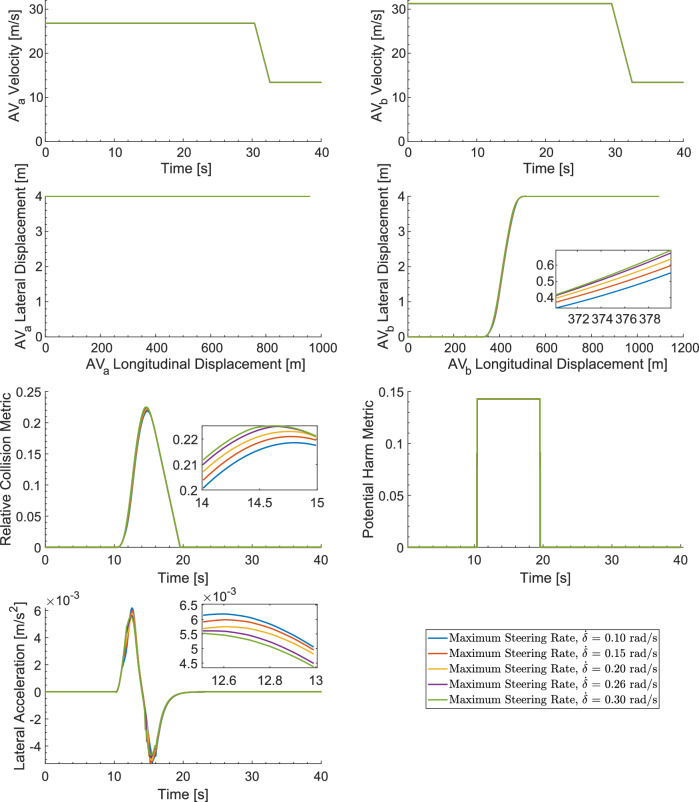
Sensitivity Analysis Results for Maximum Steering Rate

The results of the sensitivity analysis for the maximum steering rate of change, set against a fixed ML VB (i.e., $T = 1.00$, medium trust), are presented in Fig. 8. The simulation model is now utilised to explore the user experience under AV operations that employ deontological algorithms with a variable TCS related to the maximum steering rate. Steering rates ranging from 0.10 $rad/s$ and 0.30 $rad/s$ are applied in 0.05 $rad/s$ increments, representing levels from no trust to complete trust, respectively. Key findings relating to the RCM and DRI from Fig. 8 are presented in Table 6.

**Table 6:** Sensitivity Analysis Results for Maximum Steering Rate

	Maximum Relative Collision Metric (RCM),$C$	Maximum Potential Harm Metric (PHM),$H$	Duration of Risk Imposed (DRI) [Seconds]	Peak Acceleration ${a}_{peak} [m/s]$	Positive Mean Acceleration ${a}_{mea{n}_{p} } [m/s]$
± 0.10 rad/*s* (No Trust)	0.22	0.14	9.15	0.0062	0.0036
± 0.15 rad/*s* (Little Trust)	0.22	0.14	9.15	0.0060	0.0036
± 0.20 rad/*s* (Medium Trust)	0.22	0.14	9.15	0.0058	0.0037
± 0.26 rad/*s* (Medium-to- High-Trust)	0.22	0.14	9.15	0.0056	0.0037
± 0.30 rad/*s* (Complete Trust)	0.23	0.14	9.15	0.0055	0.0038

As the variable TCS decreases, indicating maximum trust and thus the highest steering rate, the risk imposed, as expected, indicates an increased risk to the occupants. Conversely, as the TCS increases, indicating minimum trust with the lowest steering rate, the risk imposeddecreases. Adjusting the trust setting from 'no trust' to 'complete trust' leads to a slight increase in the RCM, while the DRI remains unchanged. As expected, the peak PHM remains constant, owing to the effectiveness of the AV's velocity control system; both AVs adhere to speed restrictions during overtaking and maintain their position within their designated lanes. However, it should be noted that the peak acceleration, denoted as *a*_*peak*_, diminishes with increasing trust. The positive mean acceleration, denoted $a_{{mean}_{p}}$, is also analysed, showing an increase as the trust level increases. This indicates that higher TCSs result in lower peak accelerations, but these accelerations prevail over a longer duration.

## Conclusions and Future Outlook

This paper has presented a novel approach to enhancing the safe and ethical manoeuvrability of autonomous vehicles (AVs) on highways. It has described the integration of driving rules based on deontological ethical principles with the application of virtual boundaries (VBs) for AVs. An adaptive model-predictive control (MPC) algorithm, in conjunction with a dynamic bicycle model, has been utilised to model each AV and to facilitate the tracking of desired trajectories. The study has introduced a methodology for continuous risk evaluation, grounded on the interaction between two AVs. The findings demonstrate how the risk imposed can be effectively integrated into a novel user specified variable trust control setting (TCS) for use on AVs. It can be observed that increased user trust from 0 to 100%, thereby reducing the VB lengths of the AVs, results in an increased value of a relative collision metric (RCM) and an extended duration of risk imposed (DRI). This observation suggests that the variable TCS could empower AV users to feel more in control, enabling them to engage more fully with the technology. This engagement is anticipated to foster greater confidence and better acceptance of the technology as it continues to evolve.

Adoption of a user-centric scheme for AVs, such as the variable TCS proposed here, is considered to represent a stepping-stone towards enhancing the overall comfort and perceived safety of AVs during their early release phase. However, as AV users become more confident with the technology, they are likely to become less risk averse and it is anticipated that the variable TCSs will allow closer running of AVs as the technology matures.

While the results are promising, they also indicate significant opportunities for further research. Future work could include extending the simulation environment to incorporate dynamically changing conditions, which would offer a more realistic approach to modelling. Additionally, employing high-fidelity proprietary tools, such as, e.g., CarMaker, could facilitate the implementation of developed algorithms in real-time scenarios. This would allow for more comprehensive testing and refinement of the control strategies under a wider variation of realistic driving conditions, thus further advancing the field of AV technology research.

## Data Availability

Data can be provided upon request.
